# Impact of social media-supported flipped classroom on English as a foreign language learners’ writing performance and anxiety

**DOI:** 10.3389/fpsyg.2022.1052737

**Published:** 2023-01-05

**Authors:** Xiangfeng Zhao, Yanping Yang

**Affiliations:** ^1^School of Western Languages, Mudanjiang Normal University, Mudanjiang, Heilongjiang, China; ^2^Department of Management, Ordos Institute of Technology, Ordos, China

**Keywords:** flipped classroom, writing performance, anxiety, EFL, quasi-experimental research

## Abstract

As flipped classroom has received much attention from researchers and educators, some scholars have investigated the effectiveness of this teaching mode in various English as a foreign language (EFL) settings; however, such an instruction mode has been under-investigated in the Chinese EFL context. Therefore, the current study examined a flipped course’s impact on Chinese EFL learners’ writing performance and anxiety utilizing a pretest-posttest non-equivalent group quasi-experimental design. First, it selected a sample of 50 Chinese EFL learners from two intact language school classes as the participants *via* the convenience sampling method. Then, it randomly assigned participants of these two intact classes to a control group (*n* = 24), taught based on the traditional method of writing instruction, and an experimental group (*n* = 26), instructed based on social media-supported flipped instruction. The study used two writing tasks and a writing anxiety inventory to gather the data from the participants. The descriptive and inferential statistics results showed that the experimental group—taught based on flipped writing instruction—significantly enhanced their writing performance. Moreover, they revealed that the flipped course substantially reduced participants’ writing anxiety. Implications of such findings have been elaborated for EFL research and practice.

## Introduction

Although literacy development is a universal goal for all students, this aim seems to be more formidable and challenging for EFL learners, especially as far as writing skill is concerned (Yu and Lee, 2014; Wu X. et al., 2020). As the world has become text-centric, it is of much importance to improve the writing competencies of learners, a situation which makes instructors in great need of more effective techniques for writing development (Klimova, 2014; Rahimi and Fathi, 2021; Yu et al., 2021). Writing is considered as a unique skill since it requires adequate practice and knowledge of three other language skills, namely listening, reading, and speaking (Hao, 2016; Chuang et al., 2018). Additionally, writing skill needs the mastery of other competencies, including metacognitive ones (Klimova, 2014). While engaged in writing activities, learners need to establish a clear objective, plan carefully, reflect on how to organize as well as structure their writing, and revise it effectively. As reported by Walsh (2010), it is essential to learn how to write in a second language (L2) because this skill is used extensively in higher education and in workplaces. As such, in order to communicate effectively with instructors, employers, peers, or others, students need to know how to express themselves in written mode (Ferris, 1999).

For a long time, L2 researchers have sought to develop effective methodologies for teaching L2 skills. The novel and effective methods should keep up with the changing needs of students and motivate students to engage in more collaborative and individual activities inside and outside the classroom (Klimova, 2014; Hao, 2016; Wu et al., 2017; Fathi and Rahimi, 2020). A flipped classroom is an innovative teaching procedure that has caught the attention of researchers and educators particularly in the EFL contexts (Tseng et al., 2018; Jiang et al., 2022). Unlike traditional classes, where lectures precede class activities, flipped classroom is a method in which students are engaged in doing assignments inside the class and study the content themselves before coming to the class (Bergmann and Sams, 2012; Chuang et al., 2018; Wang et al., 2018; Sit and Guo, 2019; Zou and Xie, 2019; Fathi and Rahimi, 2020; Yang and Chen, 2020). As an innovative teaching method, flipped instruction reverses the order of completing assignments and classroom activities. In a traditional classroom, students gain knowledge and information mainly through teachers’ lectures and then carry out assignments at home and outside the class (Herrald and Schiller, 2013). However, students in a flipped classroom can cover pre-class teaching content at their own pace without feeling stressed or frustrated so that they can be fully prepared for in-class activities before coming to the class (Strayer, 2012; Leis et al., 2015; Chen Hsieh et al., 2017; Ho, 2020). As such, a greater portion of class time would be dedicated to students’ activities, which would enable students to synthesize their home-grown learning, ask more questions, collaborate on the required activities, and receive further insightful feedback (Gannod et al., 2007; Davies et al., 2013; Buitrago and Díaz, 2018; Wang et al., 2018). Despite the effectiveness of traditional lectures’ in transmitting information, they might be less effective when it comes to teaching skills, values, and personal development, which require more active participation on the part of the students (Johnson, 2006).

As a novel methodology, flipped instruction has become increasingly popular in L2 education mainly due to recent technological developments and novel educational orientations (Kim et al., 2014; Chuang et al., 2018). Numerous researchers have averred that flipped instruction can increase motivation, facilitate autonomous learning, and foster better learning through better management of cognitive load (Ferreri and O’Connor, 2013; Engin, 2014; Chen Hsieh et al., 2017; Tseng et al., 2018; Jiang et al., 2022). Based on this method, instructional materials are given to students prior to their class attendance, leading to their having further time to learn outside the class (Chen Hsieh et al., 2017). Consequently, learners in class are more actively engaged in the learning process as they do assignments in lieu of formal class time and learn collaboratively (Mok, 2014; Chuang et al., 2018; Fathi and Rahimi, 2020). The L2 writing process is usually accompanied by numerous challenges, including cognitive and linguistic engagement as well as lack of adequate proficiency in lexical knowledge, grammar, cohesion, and coherence (Ferreri and O’Connor, 2013; Xu and Qi, 2017). It is argued that flipping writing classrooms can alleviate such challenges and difficulties by devoting further time to doing tasks inside the classroom (Leis et al., 2015; Arifani et al., 2020; Fathi and Rahimi, 2020; Liu et al., 2022). Additionally, flipping writing instruction has been effective in improving both the global and local writing abilities of EFL learners (Wu et al., 2019; Liu et al., 2022). Also, if learners are trained to address the writing issues related to their writing content, organization, and vocabulary use during their collaborative writing activities inside the class, they will be able to provide and address peer writing mediations, which will eventually result in improving writing skills (Liu et al., 2022).

As highlighted by Aydın (2008), anxiety is an affective variable that has a significant impact on learning a foreign language (FL). For many years, L2 researchers have focused on anxiety’s role in L2 learning (Horwitz et al., 1986; Sahoo and Sinha, 2020; Gok et al., 2021). When students are highly anxious about writing, they are less efficacious and use self-regulation strategies less often than students with lower anxiety levels (Stewart et al., 2015; Paul et al., 2021). Moreover, it is argued that anxiety can pose many difficulties for foreign language students because it can hinder the acquisition, maintenance, and production of the new language (MacIntyre and Gardner, 1991). Additionally, students’ motivation, attitudes, and anxiety are all factors influencing their success in FL classes (Aydın, 2008; Tallon, 2009).

Despite the existence of some studies regarding the effects of flipped instruction on anxiety in other language skills (Gok et al., 2021; Rajabi et al., 2021), there is little research on foreign language writing anxiety. Moreover, although flipped instruction is widely researched in EFL contexts (Jiang et al., 2022), its real implementation in writing classrooms might require further exploration especially in Chinese EFL context. Therefore, the major aim of this study was to investigate the impact of flipped classroom on L2 language writing performance and writing anxiety of Chinese EFL learners using a quasi-experimental design. In this study, the flipped instruction is supported by the use of social media (e.g., YouTube, Facebook, and Instagram), which have been claimed to be useful in enhancing L2 learning (Barrot, 2021). Social media can transcend the conventional instructional constraints by meeting various needs of L2 learners (Barrot, 2018, 2020). They are also argued to be effective in developing communicative competence, cultural awareness, and other skills and components (e.g., Vásquez, 2014; Reinhardt, 2019). However, some researchers have doubted the use of social media for educational purposes as far as L2 learning is concerned (Wang and Vásquez, 2014; Aloraini, 2018; Hsu and Beasley, 2019). Therefore, two research questions are formulated to address the research objectives of this study:

Research Question 1: Compared to a conventional classroom, how effective is social media-supported flipped classroom of TOEFL training in developing Chinese adult EFL learners’ writing performance?

Research Question 2: Compared to a conventional classroom, how effective is social media-supported flipped classroom of TOEFL training in reducing Chinese adult EFL learners’ writing anxiety?

## Literature review

### Self-determination theory

Self-determination Theory (SDT) is basically a humanistic theory which posits the inherent individuals’ inclination in getting involved in their context, overcoming setbacks, and accomplishing objectives (Deci and Ryan, 1985). According to this theory, motivation types fall within a continuum based on their self-determined or relative causality locus (Deci et al., 1991). Self-determined actions have internal locus of causality while actions which are not self-determined are perceived to have external locus of control. From this perspective, motivated behavior can be self-determined if it addresses basic, inherent psychological needs of human beings including *competence*, *autonomy*, and *relatedness*.

Intrinsic motivation is the most self-determined motivation type in that if individuals are intrinsically motivated, they are devoted to doing that action because of inherent enjoyment of that activity. On the other hand, if people are extrinsically motivated, they do an activity because of its contingencies rather than the activity itself. In SDT, amotivation is also viewed as a situation in which learners have no particular intention in doing something, indicating the presence of neither intrinsic nor extrinsic motivation types. One primary concern within SDT is to explore how contextual variables can affect intrinsically motivated behaviors (Ryan and Deci, 2000).

### Flipped classroom

Flipped classroom is relatively a new concept, but it has certainly gained a lot of attention in recent years due to the incredible advances in technology devices and global access to computers and smartphones (Bergmann and Sams, 2012; Liu et al., 2022; Mirzaei et al., 2022). The traditional classroom activities, which were conducted by the teacher and homework assignments that were completed in the teacher-oriented classroom, are now carried out at home in a student-centered learning context (Davies et al., 2013; Mehring, 2016). Flipped teaching has attracted many L2 instructors recently and the idea of flipped classroom came up by many instructors dissatisfied with the boredom of classical teacher-centered classrooms as well as learner passivity in technologically laden ‘smart’ classrooms (Mehring, 2016; Tseng et al., 2018; Ho, 2020; Luo et al., 2020; Mirzaei et al., 2022). In addition to flipped classroom model (Bergmann and Sams, 2012), SDT (Deci and Ryan, 1985) can act as the theoretical underpinning of this study. According to SDT, individuals’ motivation can be explored in various educational settings in which they can meet three intrinsic psychological needs including autonomy, relatedness, and competence (Ryan and Deci, 2000). Fulfilling these needs can affect learners’ degree of motivation, engagement, and cognitive functioning. Conversely, neglecting these needs can negatively influence learners’ well-being, motivation, and learning process (Cheon and Reeve, 2015). From this perspective, flipped classroom provides the learners with a sense of autonomy, which in turn can affect their motivation, engagement, and learning outcomes.

Concerning the empirical background, several more illustrating studies investigating practices for flipping the L2 classroom are reviewed here. For example, Lee and Wallace (2018) explored the utility of the flipped instruction at a South Korean university. The participants of this study were 79 students. A number of 39 students were assigned to the group which received communicative language teaching approach, while the other 40 students were assigned to the flipped instruction group. The data were collected through the use of surveys, tasks, and teacher’s notes. The results showed that the students who received flipped instruction performed much better on the final examination than the non-flipped group. Moreover, the results indicated that participants enjoyed flipped instruction better and were more involved in the learning process. In another study, Adnan (2017) investigated the impact of flipped classroom and non-flipped classroom on EFL students’ perceptions. The data were collected according to students’ grades, journals, and interviews. The results of this study indicated that students’ perceptions of flipped classroom were generally positive, and they performed better on writing than the non-flipped instruction group. Similarly, Chen Hsieh et al. (2017) carried out a mixed methods study to explore the benefits of flipped classroom. The participants of this study were English major students. These participants were supposed to learn idioms traditionally and also with the help of LINE application. Data collection was done by the use of pre-and post-tests, questionnaires, the teachers’ observations, and semi-structured interviews. The findings indicated that by implementing flipped instruction, students’ motivation and involvement in learning increased and their knowledge of idioms improved.

In another study, Bishop and Verleger (2013) reported different but still positive attitudes toward flipped classroom by their participants. Similarly, Davies et al. (2013) explored the effect of flipped classroom on students’ achievements and the findings of this study revealed that although students were satisfied with the flipped classroom, their grades remained stable. It is generally accepted that flipped classroom has positive effects on L2 development in many aspects, as evidenced by some L2 studies reviewed above (e.g., Adnan, 2017; Chen Hsieh et al., 2017; Lee and Wallace, 2018).

Some researchers, however, maintain that some factors may contribute to success or failure of flipped instruction in engaging learners (Hao, 2016; Yilmaz, 2017; Liu et al., 2022). Students’ technological literacy is an important factor in engaging them in online classrooms. For example, Yilmaz (2017) investigated the impact of technology literacy of undergraduate students on their motivation in flipped classroom. The data were collected through some technology readiness scales, satisfaction scales, and questionnaires. The results showed that learners’ technology literacy was a crucial predictor of their motivation in flipped classroom. Therefore, the learners must be prepared to conduct classes in an online environment, work with different apps, and communicate in a new environment. Additionally, Hao (2016) claimed that learning in the flipped class requires responsibility and commitment as well as some basic technical skills for students to be able to manage a class effectively. As a result, one of the variables affecting the success of flipped instruction is the ability to manage one’s own learning in technology-enhanced learning environments.

### Flipped classroom for writing skill

Writing is an important skill for L2 language acquisition and is considered an essential communication tool in the modern world. In a typical writing classroom, EFL students often struggle to write due to confusion about grammar, limited vocabulary, and a lack of knowledge about how various genres of writing work (Chen, 2002). For students with low proficiency, writing skill is particularly difficult as they are restricted in terms of resources, processes, and controls (Chenoweth and Hayes, 2003). As a result, these students lack the resources and have trouble controlling their writing for genre suitability, accuracy, and fluency (Su Ping et al., 2020). Consequently, teaching low proficiency students how to compose appropriate texts could be even more challenging (Su Ping et al., 2020). A majority of EFL teaching methodologies fail to develop writing skill or neglect the abilities required for L2 writing (Wu V. W.-C. et al., 2020; Mirzaei et al., 2022). Despite the fact that intermediate level L2 students often feel dismayed by the lack of skills they possess in expository writing, college and university programs provide many remedial courses for L2 writers in order to help them overcome the inflexible cognitive demands of the writing skill (Awada et al., 2020). In this direction, a significant number of studies have addressed the positive impact of flipped classroom on students’ writing performance (Alghasab, 2020; Judy Shih and Huang, 2020; Su Ping et al., 2020). For example, Alghasab (2020) investigated the impact of flipped classroom on EFL students’ writing skill. Data collection was done by the use of questionnaires and semi-structured interviews. The findings of this study revealed that students held positive perceptions of flipped classroom. Moreover, the flipped classroom environment provides students with useful learning opportunities alongside an increase in their motivation and communication. Additionally, Judy Shih and Huang (2020) carried out a qualitative research design to explore the effect of flipped classroom on EFL students’ metacognitive strategies. The participants of this study were eight EFL students and the data were collected by the use of semi-structured interviews. The results indicated that flipped classroom allowed students to employ metacognitive strategies in a more engaging and effective manner. In another study, Su Ping et al. (2020) investigated the EFL learners’ attitudes toward flipped classroom. Data were collected using semi-structured interviews and the results revealed that flipped classroom provided students with further time, further practices, interactive discussions, motivation, and instant feedback during class time. Consequently, students’ attitudes were positive towards flipped instruction. These findings were also confirmed by Fathi and Rahimi (2020) who reported the effectiveness of flipped classroom in affecting writing performance of EFL learners.

### Social media in L2 learning

As the Internet has induced a technology revolution and contributed to the ever-dominance of communication technologies for sharing information, social media have emerged as the affordable technology devices, leading to the more convenient access to information. Social media can be characterized as ‘web-based or personal device-based applications that connect users with online resources’ (Evans, 2014, p. 903). Social media, which are the Internet-dependent technology devices, can foster learners’ engagement and cognitive development (Santovena-Casal and Bernal-Bravo, 2019). Adopting the Manca’s (2020) conceptualization of social media in the educational contexts, the present researchers considered social media devices as Internet-based applications for sharing images (e.g., Instagram), video sharing applications (e.g., YouTube), and instant messaging applications (e.g., WhatsApp). Numerous researchers have verified the utility of social media in L2 learning (e.g., Kukulska-Hulme and Viberg, 2018; Reinhardt, 2019; Aloraini and Cardoso, 2020; Yeh and Swinehart, 2020; Barrot, 2021; Roohani and Heidari Vincheh, 2021). It is worth noting that social media is a vehicle for implementing flipped instruction in the present study.

### Writing anxiety

Researchers have found that flipped classrooms could have a significant impact on L2 learning skills, including speaking and writing in addition to academic success (Boyraz and Ocak, 2017; Tseng et al., 2018; Sit and Guo, 2019; Fathi and Rahimi, 2020; Jiang et al., 2022). Because L2 writing is so demanding and requires numerous language competencies like task achievement, coherence and cohesion, vocabulary, grammatical range, accuracy, it creates a high level of writing anxiety (Baepler et al., 2014; O’Flaherty and Phillips, 2015; Fareed et al., 2016). Generally, *writing anxiety* is defined as an aversion to writing and situations in which some amount of writing is perceived by the individual, along with the potential for evaluating that writing (O’Flaherty and Phillips, 2015; Fareed et al., 2016). This avoidance is likely to lead to a fear of the writing process outweighing the projected benefits of writing ability (Thompson, 1980; Fathi and Khodabakhsh, 2020). Since L2 writing entails complex cognitive processes that can cause frustration and difficulty for L2 students at any proficiency level, L2 writers perceive it as a particularly challenging language skill (Baepler et al., 2014; Fareed et al., 2016). Writing anxiety has been considered by a variety of researchers as a multidimensional construct encompassing several dimensions. As revealed by Rankin-Brown (2006) writing anxiety involves the frustration of assessing one’s own writing quality and comparing it to one’s expectations, the fear of teacher’s feedback, the fear of peer feedback, and the fear of losing one’s identity. A person with writing anxiety is likely to have apprehensions and unpleasant feelings that can adversely affect the writing process (Zheng and Cheng, 2018). When completing L2 tasks, learners with writing anxiety often experience low self-esteem, apprehension, tension, procrastination, avoidance, and withdrawal (Cheng, 2004). It is argued that writing anxiety is caused by an individual’s writing ability, readiness to complete the writing task, and fear of being judged or evaluated by others (Cheng, 2004). In addition, Cheng (2004) considered L2 writing anxiety to be a three-dimensional construct. Based on the physiological, cognitive, and behavioral aspects of writing anxiety as well as how these dimensions influence L2 writing outcomes, Cheng (2004) claimed that L2 writing anxiety is rooted in the belief that anxiety experiences like nervousness and tension have physiological effects, negative emotions about others’ expectations, and avoidance to perform a particular behavior. According to other studies (e.g., Rankin-Brown, 2006), some more factors can cause L2 writing anxiety such as low self-esteem, limited linguistic proficiency, and low self-efficacy. Given the importance of writing anxiety in L2 learning, some researchers have argued that flipped classroom might be an effective teaching method which can alleviate this type of apprehension in L2 context (Xu and Qi, 2017; Tseng et al., 2018).

## Materials and methods

### Study design

This study is a quantitative, quasi-experimental research. As the participants were the students of two intact classes and it was not possible to select and divide the participants randomly in this language school, the researchers employed a quasi-experimental pretest-posttest non-equivalent groups design. This research design is normally used in contexts where it is not logistically feasible to conduct a randomized, controlled trial research (Ary et al., 2018). In this design, the researchers compared the performance of an experimental group and a control group on two measures (i.e., writing performance and writing anxiety) gathered before and after the experiment.

### Participants

In order to accomplish the aims of this quasi-experimental study, a sample of 50 Chinese EFL learners from two intact classes of a language school in Hunan province, China were selected as the participants. Convenience sampling method was employed to select these participants since this sample was more available for the research team. The participants of these two intact classes were assigned to a control group (*n* = 24) and an experimental group (*n* = 26). The sample was comprised of both male and female EFL learners whose ages varied from 19 to 24 (*M* = 21.62, SD = 3.92). This language school was primarily concerned with preparing the upper-intermediate Chinese EFL learners to get ready for the TOEFL exam. More precisely, the purpose of this course that the two classes had enrolled in was to increase writing skills of the TOEFL applicants *via* an intense writing course. The students had previously studied English during their school days. According to the students’ self-report, the students had not previously experienced being taught based on flipped writing instruction. The two classes were taught by a 35-year-old male English teacher who cooperated with the researchers in accomplishing the objectives of this study. The teacher was a trained TOEFL instructor who had the experience of using technology and flipped instruction in his previous courses. After obtaining his informed consent, the researchers provided him with the general explanation and guideline of the purpose of the research and the procedures he needed to employ in each class.

### Study variables

In this research, the independent variable was the mode of instruction, with two levels (i.e., flipped classroom or conventional classroom), a categorical variable. Writing performance and writing anxiety were the two dependent variables under investigation. The former was measured with timed writing tasks and the latter was evaluated by administering a previously designed Likert-scale questionnaire of L2 writing anxiety.

### Instruments

#### Language proficiency test

Because the general language competence of the participants could affect their writing skill, participants overall English proficiency was measured *via* administering Oxford Placement Test (OPT; Allan, 2004). OPT is characterized as a reliable and valid measure of English proficiency widely used as a placement test for various learners of English (Allan, 2004). The mean scores of the two groups obtained from OPT were compared by running an independent samples *t* test whose results showed no significant difference between the two groups, confirming the homogeneity of the participants with regard to general English proficiency. The internal consistency of OPT, as estimated by Cronbach’s alpha, was 0.82 in this study.

#### Timed writing tasks

In order to measure the writing performance of the Chinese EFL students, two 40-min writing tasks were utilized as the pre-test (Task 1) and post-test (Task 2) of this research. The reason for using two separate writing tasks for pre-and post-tests was to avoid test effect as the potential internal validity threat (Ary et al., 2018). The topics of the timed tasks were selected from sample TOEFL writing essays and did not need specialized schematic knowledge. The participants were requested to write at least 400 words for each task. The two tasks were as the following:

Task 1: Some individuals prefer to get up early in the morning. Others tend to get up later in the day. Which one do you agree with? Use specific reasons to justify your choice.
Task 2: Some individuals prefer to travel with a companion. Others like to travel alone. Which one do you agree with? Use specific reasons to justify your choice.

The completed writing tasks of the participants were marked using Jacobs et al.’s (1981) rubric which is based on an analytical scoring method. This writing scoring rubric evaluates the quality of written texts in terms of content, language use, organization, vocabulary use, and writing mechanics. The inter-rater reliability was assured by inviting two other trained raters for scoring the timed writing tasks independently. Cohen’s Kappa’s formula was used and it demonstrated an acceptable level of rater consistency (*ɑ* = 0.83).

#### Writing anxiety inventory

English as a foreign language students’ writing anxiety was gauged using Cheng’s (2004) L2 Writing Anxiety Inventory. This inventory includes 22 statements assessing three components of *somatic anxiety*, *cognitive anxiety*, and *avoidance behavior*. Each statement is evaluated on a 5-point Likert scale from 1 (*strongly disagree*) to 5 (*strongly agree*). This scale was given to the participants of the two groups twice, as pre-and post-tests. The reliability coefficients of the scale were 0.83 and 0.86 before and after the treatment, respectively.

### Experimental procedure and data collection

As discussed above, the purpose of the course, which lasted for about 3 months, was to help EFL students to improve their writing abilities by practicing to write various types of paragraphs and essays. To this aim, the instructor asked the students to do various writing tasks regarding different kinds of paragraphs such as comparison and contrast, expository, argumentative, process, definitional, and narrative paragraphs. Also, the EFL learners were taught how to outline an essay, develop its different sections (e.g., introduction, body, and conclusion), and revise the written texts. The same textbook and course materials were used for both groups who were instructed by the same teacher. The experiment began in late March of 2022 and was finished in early June. There were equal 16 sessions of procedure for each group, two of which (i.e., the first and the last sessions) were devoted to administering pre-and post-tests.

#### Micro-lecture videos and their delivery method

Concerning the specific purpose of the study, the researchers used Bergmann and Sams’s (2012) model of the flipped classroom. Based on this model, the experimental group were provided with previously created/prepared videos and clips taken from Internet, especially YouTube, or selected from the previously held online classes of the instructor. Some clips were also adapted from social media such as Instagram, Facebook, and WhatsApp/Telegram Channels. The duration of video clips ranged from 15 to 30 min. The students were given clips before each session and were required to watch them and do the worksheet exercise before attending the class. The worksheet completion by the flipped group could indicated that the students have watched the videos before coming to the class.

#### In-class activities

Each session of the experimental group lasted about 90 min. During the class time, the instructor first ensured that the students had covered and understood the content of the video clips by checking students’ worksheets or asking some questions (20 min). Afterwards, the students were involved in group discussions related to the content of the video clips in addition to doing the corresponding exercises of the textbooks collaboratively (60 min). In fact, the students were divided in small groups for the collaborative completion of the tasks. At the end of each session, the students were provided with a brief introductory explanation of the topics/tasks for the next session (10 min). As the students were exposed to videos in advance, the flipped group had further time to do in-class activities and receive peer and teacher feedback.

#### Non-flipped group

**Figure 1:**
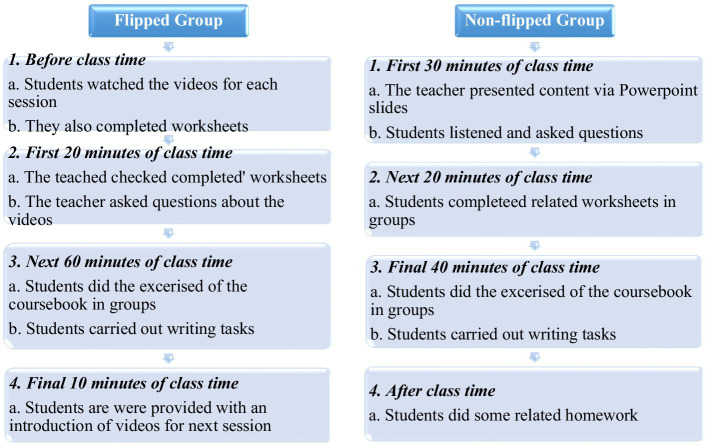
Instructional procedures of flipped and non-flipped groups.

On the other hand, the control group students received the same instructional materials and tasks that the flipped group did. However, no videos or materials were given to the students prior to the class. Each session of the non-flipped group also lasted for about 90 min. First, the content of videos was explained by the teacher *via* lectures or PowerPoint presentations (30 min). After that, like the flipped group, the non-flipped class collaboratively completed the corresponding worksheets of each session (20 min). Finally, the students of the non-flipped group were also engaged in doing the same writing tasks and exercises inside the classroom collaboratively (40 min). They also wrote the essays after experiencing the sequential stages of rehearsing, drafting, redrafting, and revising. Since the non-flipped group were not exposed to the videos and class contents, they had less time (i.e., 20 min less than the time of the flipped group) to practice or do written tasks compared to their counterparts in the flipped group. As a result, the number of completed tasks or written assignments in the control group was fewer than those in the experimental group. The instructional procedures of flipped and non-flipped groups is presented in Figure 1.

#### Teaching materials

The main coursebook was Lougheed’s (2008) book entitled *Barron’s Writing for the TOEFL IBT: With Audio CD*. This book intended to enhance writing skills of the students to get better scores on writing tasks. The manual was structured simply in a step-by-step fashion to help EFL learners to develop ideas, organize details, and write them into vivid, well-organized English. The book also provided the learners with model essays and writing exercises to get more familiar with well-structured writing tasks.

### Data analysis

The gathered data were fed to SPSS (version 22.0) for statistical analysis. Both descriptive statistics (means and standard divisions) and inferential statistics were taken into consideration. Concerning the inferential statistics, paired samples t-tests and the analysis of covariance (ANCOVA) were performed to analyze the effect of the independent variable on the two dependent variables. According to Pallant (2020), ANCOVA can be used when there is a pre-test/post-test design in which the pre-test scores are regarded as the covariate. In this analysis, the independent variable was the teaching type (i.e., flipped or traditional) and the dependent variables were writing performance and writing anxiety scores measured at the end of experiment. As required by ANCOVA analyses, some preliminary checks were made to ensure that the assumptions were all met. For this purpose, the assumptions of linearity, normality, homogeneity of variances, homogeneity of regression slopes, and reliable estimation of the covariate were all checked.

## Results

Concerning the data analysis for the investigation of the impact of a social media-supported flipped L2 writing course on the writing performance and writing anxiety of the EFL participants, both descriptive (mean and standard deviation) and inferential statistics were taken into account. In addition to paired samples t-test, ANCOVA was conducted to identify the effects of the flipped instruction on the writing skill and writing anxiety of EFL learners. In fact, two ANCOVAs were performed to explore the effect of flipped classroom on the two dependent variables of writing performance and writing anxiety. The pre-test scores were considered as the covariates and the posttest scores were the dependent variables in the analyses.

As far as the effect of social media-supported flipped classroom on the writing performance was concerned, the descriptive statistics demonstrated that the mean score of the experimental group underwent a bigger increase than the control group. As shown in Table 1, the mean score for the writing performance of the experimental group increased from 52.66 (SD = 10.5) on the pre-test to 69.12 (SD = 11.1) on the post-test. Likewise, the mean score of the control group rose from 51.08 (SD = 9.8) on the pre-test to 60.13 (SD = 10.1) on the post-test. As indicated in Table 2, the results of paired samples t-test also indicated that there was a significant change in the writing mean scores of the experimental group (*t* = −4.2, *p* < 0.01, *d* = 0.52). Likewise, the increase in the writing mean scores of the control group was significant (*t* = −2.3, *p* < 0.05, *d* = 0.33).

**Table 1:** Descriptive statistics of the two groups.

Groups	Scales	Pre-test		Post-test	
		*M*	SD	*M*	SD
Experimental	Writing skill	52.6	10.9	69.1	11.1
	Writing anxiety	38.8	7.3	25.9	5.7
				
Control	Writing skill	51.0	9.8	60.1	10.1
	Writing anxiety	37.0	6.9	32.1	7.3

**Table 2:** Paired samples *t*-test results, examining the differences between the pre- and post-tests of the two groups on the writing performance.

	Pre-test		Post-test				Cohen’s *d*
	*M*	SD	*M*	SD	*t*	*p*	
Experimental	52.6	10.9	69.1	11.1	−4.2	0.000	0.52
Control	51.0	9.8	60.1	10.1	−2.3	0.022	0.33

However, ANCOVA results verified that there was a significant difference between the two groups on post-test mean scores of writing performance, *F*(1, 48) = 10.12, *p* = 0.000, partial eta squared = 0.21; as seen in Table 3. This outcome showed that the EFL students of the experimental group improved their writing performance substantially more than the control group participants, confirming that the social media-supported flipped writing instruction contributed to enhancing the writing competencies of the EFL students.

**Table 3:** ANCOVA results, investigating the effect of flipped classroom on the EFL students’ writing performance after controlling for the covariates.

Source	Type III Sum of Squares	*df*	Mean square	*F*	Sig.	Partial Eta squared
Covariate (pre-test)	3.22	1	3.22	0.07	0.86	0.00
Between-subjects	526.00	1	526.00	10.12	0.000	0.21
Within-subjects	2152.02	48	52.35			

The second purpose of the research was to explore the effect of flipped teaching on the L2 writing anxiety of the EFL participants. As seen in Table 1, the descriptive statistics shows that the mean score of the L2 writing anxiety of the experimental group decreased from 38.87 (SD = 7.37) on the pre-test to 25.92 (5.72) on the post-test. By the same token, the mean score for the control group decreased from 37.01 (SD = 6.59) on the pre-test to 32.16 (SD = 7.38) on the post-test of L2 writing anxiety.

In addition, as presented in Table 4, the results of paired samples t-test demonstrated that a significant change was found in the writing anxiety mean scores of the experimental group (*t* = 5.67, *p* < 0.01, *d* = 0.43). Similarly, there was a significant decrease in the writing anxiety mean scores of the control group (*t* = 2.27, *p* < 0.05, *d* = 0.19).

**Table 4:** Paired samples *t*-test results, examining the differences between the pre- and post-tests of the two groups on the on the writing anxiety.

	Pre-test		Post-test				Cohen’s *d*
	*M*	SD	*M*	SD	*t*	*p*	
Experimental	38.87	7.37	25.92	5.72	5.67	0.001	0.43
Control	37.01	6.95	32.16	7.38	2.27	0.031	0.19

Moreover, ANCOVA was performed by adjusting for the pre-test scores of writing anxiety. The results (as shown in Table 5) demonstrated that a substantial difference was found between the mean scores of the two groups in terms of writing anxiety, *F*(1, 48) = 7.12, *p* = 0.003, partial eta squared = 0.18). This outcome indicated that social media-supported flipped classroom was useful in reducing the writing anxiety of the Chinese EFL learners.

**Table 5:** ANCOVA results, investigating the effect of flipped classroom on the EFL students’ writing anxiety after controlling for the covariates.

Source	Type III sum of squares	*df*	Mean square	*F*	Sig.	Partial Eta squared
Covariate (pre-test)	132.165	1	132.16	91.38	0.62	0.00
Between-subjects	10.324	1	10.32	7.12	0.003	0.18
Within-subjects	63.198	48	2.83			

## Discussion

This quasi-experimental study aimed to explore the effects of a flipped writing course on the writing performance and writing anxiety of Chinese EFL students. The results obtained from paired samples t-tests and ANCOVAs yielded significant findings. First, the findings indicated that social media-supported flipped classroom significantly improved the writing performance of Chinese EFL learners. These results lend support to the outcomes of numerous studies focusing on the effect of flipped classroom on the writing skill (Wen, 2013; Mok, 2014; Leis et al., 2015; Mehring, 2016; Arifani et al., 2020; Fathi and Rahimi, 2020; Liu et al., 2022). As reported by Fathi and Rahimi (2020), flipped classrooms developed the writing skills of EFL students better than non-flipped classrooms and one possible justification for this finding was attributed to the assignments carried out by students in flipped and non-flipped classrooms. As a result of using non-flipped instruction, EFL students did their homework independently after the class. Therefore, there was less peer or teacher evaluation and students were rarely able to evaluate their tasks. However, the EFL students in the flipped classroom had an opportunity to have further interaction with the material, their peers, and the instructor than they did in the non-flipped classroom since they could view the videos whenever they wanted and at their own pace, thereby becoming well prepared for the class activities (Mok, 2014; Mehring, 2016; Luo et al., 2020; Yang and Chen, 2020). Consequently, in the classroom, the students’ output could serve as a starting point, which would motivate them to participate in different class activities. Additionally, the flipped group had further exposure to feedback and task-oriented inputs (Wen, 2013) than the non-flipped group. This instructional procedure also helped the students to do the tasks collaboratively, and in some cases, helped them become more confident EFL learners. Moreover, it is possible that the results are influenced by the students’ high motivation and engagement in flipped classrooms (Chen Hsieh et al., 2017). According to Chen Hsieh et al. (2017), the students in flipped classrooms spent a lot of time on pre-class activities that would allow them to perform the in-class activities more successfully. Therefore, in the class, the students were fully engaged in several class activities, enabling them to perform the class tasks more effectively and meaningfully. Additionally, further assistance as well as feedback was provided to the flipped group students by the instructor. On the other hand, students in the non-flipped classroom were less involved in class activities than the students in the flipped classroom. It was because the students in the non-flipped classroom had no pre-class tasks, and thus were not fully prepared for class activities (Wen, 2013; Fathi and Rahimi, 2020). In addition, having more time to prepare for the lessons before class and receiving immediate feedback from the instructor and peers during class could have motivated the EFL students to write more effectively in class (Su Ping et al., 2020). When the students were discussing the topics, sharing ideas, and practicing writing together, they felt more engaged and interested in class activities. Overall, students of the flipped group might have felt better and gained high levels of confidence and motivation in writing, resulting in their better writing performance at the end of the course.

The second purpose of this study was to explore the impact of social media-supported flipped classroom on writing anxiety of EFL learners. The results showed that flipped instruction effectively reduced EFL students’ writing anxiety. As mentioned by some researchers (e.g., Thompson, 1980; O’Flaherty and Phillips, 2015; Fathi and Khodabakhsh, 2020), writing anxiety is an aversion to writing and it is likely to lead to a kind of fear in the writing process. This fear can hinder students from learning to write. *Via* flipped instruction, students are likely to feel less pressure to rush and they could do their writing homework at home more peacefully and conveniently. On the other hand, shy students in traditional classrooms might be worried about their writing outcomes evaluated by the teacher and peers (Wen, 2013), as a result, they prefer not to write instead of making possible mistakes. Moreover, in the traditional classrooms, the required time for doing the writing tasks is stable for all the students, neglecting the fact that some students might require more time for brainstorming than others. However, by the use of flipped instruction, students’ individual differences are taken into account, contributing to decreasing their writing anxiety. As a result of using flipped instruction and being fully prepared for the class, students’ inspiration, self-awareness, and self-confidence are enhanced, all of which might have helped in reducing writing anxiety of the experimental group students. Taken together, the findings can be justified in light of SDT (Deci and Ryan, 1985) by positing that flipped classroom might have provided the Chinese EFL learners with a sense of autonomy, which in turn could have influenced their motived behavior in L2 writing, resulting in their greater engagement, better writing, and reduced anxiety. Also, from a broader perspective, the findings of this study, which revealed that social media-supported flipped learning was effective to be employed for EFL writing classroom, can support the extant literature (e.g., Rosell-Aguilar, 2018; Barrot, 2020, 2021) evidencing the benefits of using social media for L2 learning.

## Conclusions and implications

The findings of this study offered empirical support for the effectiveness of flipped instruction in an EFL context in general and in wiring classrooms in particular. The findings suggest several implications for EFL researchers and instructors. First of all, social media-supported flipped instruction might have some merits for EFL students especially for EFL writing courses as it was found to be effective both in enhancing writing skill and in decreasing writing anxiety of EFL students. Second, this mode of instruction gives a sense of direction to instructors by helping them not only plan and prepare materials for the class, but also scaffold students’ learning during the class. Third, given the detrimental effects of L2 anxiety, students should be taught how to deal with anxiety, or the teacher can create a less distracting environment in the classroom. Flipping the classroom can create a less stressful classroom environment in which assignments are not delegated and students can study in a learner-centered environment alongside their peers and teacher. Although the instructor maintains the role of director and students interact with the teacher in a formal setting during the class time, the flipped classroom allows the teacher to serve both as facilitator as well as an observer, resulting in greater engagement between students and the instructor. As a result, flipped classrooms offer a flexible environment conducive to reducing anxiety and boosting students’ self-esteem and performance. Having been taught based on flipped instruction, EFL students with low proficiency levels might become more motivated to write since they have more time to prepare for lessons before class and receive immediate feedback from their instructor and peers during class, which can scaffold their learning. In addition, students need to understand the rationale behind flipped classroom in order to engage effectively in class activities, and instructors can improve students’ participation by creating motivation and encouragement.

This study has provided a better understanding of the impact of social media-supported flipped classroom on L2 writing performance and writing anxiety of EFL learners, but further research is still required to reach more conclusive results. As such, some limitations can be mentioned in this regard. First, the researchers employed quantitative research method to address the research questions in this study. Therefore, future researchers can gather qualitative data to clarify the effect of flipped classroom in EFL contexts more vividly. Also, it is worth considering that the relatively small sample size might be a concern in this research. However, it is generally argued that the minimum number of 30 participants might suffice for quasi-experimental or *ex post facto* studies (Dornyei, 2007). In addition, it should be noted that as the sample size in L2 writing classes is usually small (Lin, 2014), the sample size of the present research seems to be acceptable. Therefore, future studies may investigate flipped classroom method and anxiety among larger samples in different learning contexts. The flipped learning research could benefit from investigating the effect of different online learning environments comparatively and how they can influence anxiety levels of the participants. Finally, employing longitudinal studies will offer deeper insight into the effect of flipped instruction on L2 learning outcomes.

## Data availability statement

The original contributions presented in the study are included in the article/supplementary material, further inquiries can be directed to the corresponding author.

## Ethics statement

The studies involving human participants were reviewed and approved by Management Department, Ordos Institute of Technology, Ordos 010071, Inner Mongolia, China. The patients/participants provided their written informed consent to participate in this study.

## Author contributions

XZ and YY carried out data analyses and wrote the manuscript. All authors contributed to the article and approved the submitted version.

## Funding

The research is supported by the Study and Practice of Innovating College English Teaching Mode and Cultivating International Talents (2018–2022).

## Conflict of interest

The authors declare that the research was conducted in the absence of any commercial or financial relationships that could be construed as a potential conflict of interest.

## Publisher’s note

All claims expressed in this article are solely those of the authors and do not necessarily represent those of their affiliated organizations, or those of the publisher, the editors and the reviewers. Any product that may be evaluated in this article, or claim that may be made by its manufacturer, is not guaranteed or endorsed by the publisher.
